# The Link Between Epigenetic Clocks for Aging and Senescence

**DOI:** 10.3389/fgene.2019.00303

**Published:** 2019-04-03

**Authors:** Wolfgang Wagner

**Affiliations:** ^1^Division of Stem Cell Biology and Cellular Engineering, Helmholtz Institute for Biomedical Engineering, RWTH Aachen University Medical School, Aachen, Germany; ^2^Institute for Biomedical Engineering – Cell Biology, RWTH Aachen University Medical School, Aachen, Germany

**Keywords:** DNA methylation, epigenetic, senescence, aging, iPSC, quality control, senolytic drugs, biomarker

## Abstract

Replicative senescence of cells *in vitro* is often considered as counterpart for aging of the organism *in vivo*. In fact, both processes are associated with functional decay and similar molecular modifications. On epigenetic level, replicative senescence and aging evoke characteristic modifications in the DNA methylation (DNAm) pattern, but at different sites in the genome. Various epigenetic signatures, which are often referred to as epigenetic clocks, provide useful biomarkers: Senescence-associated epigenetic modifications can be used for quality control of cell preparations or to elucidate effects of culture conditions on the state of cellular aging. Age-associated epigenetic modifications hold high expectations to determine chronological age in forensics or to identify parameters that impact on biological aging. Despite these differences, there are some striking similarities between senescence- and age-associated DNAm, such as complete rejuvenation during reprogramming into induced pluripotent stem cells (iPSCs). It is yet unclear what makes epigenetic clocks tick, but there is evidence that the underlying mechanisms of both processes are related to similar modifications in the histone code or higher order chromatin. Replicative senescence therefore appears to be a suitable model system to gain better insight into how organismal aging might be governed epigenetically.

## Introduction

Primary cells, which are taken directly from living tissues, can only be culture expanded for a limited number of cell divisions before entering irreversible proliferation arrest (Campisi and d’Adda di Fagagna, 2007). Since the first description of this phenomenon by Hayflick and Moorhead (1961) it has been discussed if replicative senescence is merely a cell culture artifact, or if it is directly related to aging of the organism. In fact, both processes are associated – and potentially caused – by telomere attrition (Harley et al., 1990; Allsopp et al., 1992). Furthermore, accumulating DNA damage and functional decline of mitochondria may contribute to metabolic dysfunction (Sun et al., 2016; McHugh and Gil, 2018). Additional commonalities of senescence and aging include alteration of cellular morphology, metabolic changes, loss of differentiation potential, activation of the p53/p21CIP1 and p16INK4A/pRb signaling pathways, increased senescence-associated β-galactosidase activity (SA-β-gal), formation of senescence-associated heterochromatic foci (SAHF), and the senescence-associated secretory phenotype (SASP) (Lopez-Otin et al., 2013). Cellular senescence is even considered as a hallmark of aging, as senescent cells accumulate in aged tissues (Lopez-Otin et al., 2013), but it remains to be proven if the underlying mechanisms for senescence and aging are directly related (Campisi, 2013).

The phenomenon of cellular senescence is quite diverse. Decreasing proliferation of cells in culture is not only evoked by long-term expansion, but it can also be triggered, e.g., by irradiation or oncogene induced senescence (Campisi and d’Adda di Fagagna, 2007). While these different types of cellular senescence result in similar functional and morphologic changes on cellular level, they can clearly be distinguished by molecular means. Unless stated otherwise, I will particularly focus on replicative senescence in this mini review. Furthermore, I apologize that due to space constrains for this format it will not be possible to give credit to all the important studies in this field and I will particularly discuss the possible link between DNA methylation (DNAm) changes in aging and replicative senescence.

**FIGURE 1:**
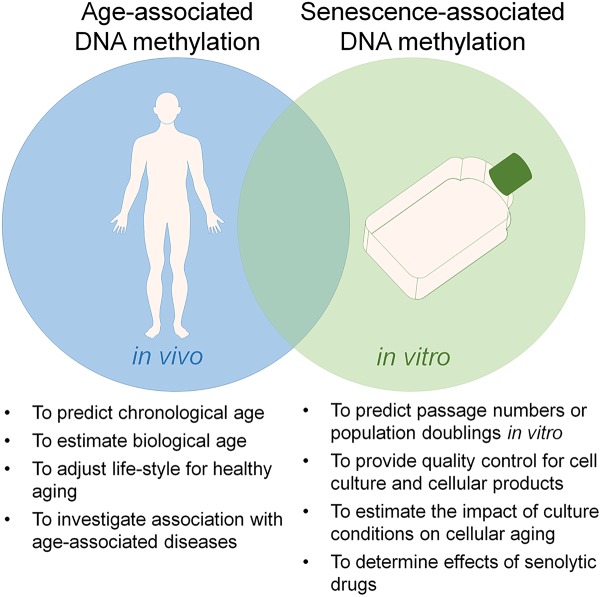
Utility of epigenetic clocks for aging and senescence. Aging and replicative senescence are both reflected by highly reproducible DNA methylation (DNAm) changes and there is a significant overlap between these epigenetic modifications. On the other hand, the two processes can be tracked independently by epigenetic signatures, which can be utilized for different applications.

There is a growing perception that culture expansion toward replicative senescence and aging are both reflected by specific modifications in the DNAm pattern, which might indicate that they are evoked by epigenetic processes (Wagner et al., 2016). DNAm plays a crucial role in mammalian development (Meissner et al., 2008). It occurs predominantly in the context of cytosine-guanine dinucleotides – so called CpG sites. The methylation pattern of CpG sites is maintained and modified by DNA methyltransferases (DNMTs) (Liao et al., 2015). On the other hand, demethylation can be mediated indirectly by ten-eleven translocation (TET) family enzymes, which oxidize 5-methylcytosine into 5-hydroxymethylcytosine that can either be passively depleted through DNA replication or actively reverted to cytosine by iterative oxidation and thymine DNA glycosylase (TDG)-mediated base excision repair (Kohli and Zhang, 2013). However, it is largely unclear how DNAm changes are regulated at specific sites in the genome. Furthermore, the functional relevance of specific DNAm changes remains largely elusive. In 2010, our group has demonstrated that aging as well as senescence are reflected by DNAm changes at specific CpG sites (Bork et al., 2010). Since then, many other groups have fine-tuned epigenetic signatures that can be used to track these processes independently and for different purposes (Figure 1), demonstrating that replicative senescence and aging are epigenetically distinct (Wagner et al., 2016; Kabacik et al., 2018).

## An Epigenetic Clock that Underlies the Hayflick Phenomenon

Almost 30 years ago, it has been suggested that cell culture is associated with a continuous loss in the general DNAm level, and that this loss might be related to the number of cell divisions (Catania and Fairweather, 1991). These authors speculated that the progressive loss of DNAm could generate a multi-step cell division “clock” which underlies the Hayflick phenomenon. The today available global DNAm profiles reveal a more complex picture. Whole-genome single-nucleotide bisulfite sequencing has demonstrated widespread DNA hypomethylation and focal hypermethylation upon replicative senescence (Cruickshanks et al., 2013). In fact, a relatively large proportion of the genome reveals reproducible and highly significant changes of DNAm during culture expansion, particularly in developmental genes (Koch et al., 2013). Furthermore, chromatin conformation analysis demonstrated that replicative senescence is associated with an unidirectional loss in local chromatin connectivity, suggesting that senescence is an endpoint of the continuous nuclear remodeling process during differentiation (Chandra et al., 2015; Criscione et al., 2016). Yet, senescence-associated DNAm changes are relatively consistent across different cell types (Franzen et al., 2018; Zirkel et al., 2018).

In our previous work, we have utilized DNAm profiles of mesenchymal stromal cells (MSCs) and fibroblasts, which at the time were analyzed with 27k Illumina BeadChip arrays, to select six CpGs that revealed almost linear hyper- or hypo-methylation with subsequent passages (Koch et al., 2012). Thus, DNAm levels at these six CpGs can be used to estimate the number of passages during *in vitro* expansion or the number of cumulative population doublings (cPDs) (Koch and Wagner, 2013). This relatively small Epigenetic Senescence Signature facilitates a targeted analysis by pyrosequencing in a fast and cost-effective manner. Tracking of the state of senescence is of particular importance for MSCs, which raise high hopes in regenerative medicine and are currently tested in a multitude of clinical trials (Wagner and Ho, 2007). In comparison to other biomarkers for senescence, such as telomere attrition (Bernadotte et al., 2016) or staining of SA-β-gal (Itahana et al., 2007), the DNAm changes provide a more quantitative measure. Notably, gamma irradiation of MSCs resulted in typical senescence-associated changes in morphology, gene expression, and loss of differentiation potential, but in contrast to replicative senescence it did not evoke any significant DNAm changes (Koch et al., 2013). This exemplifies that the different types of senescence – e.g., oncogene induced senescence, DNA damage induced senescence, or replicative senescence – seem to be triggered by different mechanisms (Koch et al., 2013; Lowe et al., 2016; Kabacik et al., 2018).

## Aging is Reflected by Specific DNA Methylation Changes

Already in 1973 it was described that aging is reflected by a global decrease in the 5-methylcytosine content of various tissues, and this led to the assumption that DNAm may regulate gene activity and somehow trigger the process of aging (Vanyushin et al., 1973) – a hypothesis that is still valid. Presently, it is estimated that almost one third of the CpG sites reveal age-associated DNAm changes, of which 60% become hypomethylated and 40% hypermethylated upon aging (Johansson et al., 2013). Overall, the age-associated DNAm changes are similar across different tissues, while they are certainly influenced by the very different epigenetic makeup of different cell types (Teschendorff et al., 2010; Koch and Wagner, 2011).

The first epigenetic clocks to estimate donor age were again derived from the 27k Illumina Bead Chips (Bocklandt et al., 2011; Koch and Wagner, 2011). These signatures were further improved in the advent of more available datasets and refined bioinformatics approaches (Hannum et al., 2013; Horvath, 2013; Weidner et al., 2014). For example, Hannum et al. (2013) used DNAm profiles of 656 whole blood samples of donors aged 19–101 and integrated 71 CpG sites into a multivariable linear regression model enabling age-predictions with an error of 4.9 years in an independent validation dataset of blood samples. In analogy, we derived similar epigenetic aging signatures for blood (Weidner et al., 2014; Lin et al., 2016). Horvath established a very robust multi-tissue predictor by an elastic net regression model that was trained on 7,844 samples from 82 datasets (including 51 different tissues and cell types) (Horvath, 2013). This model is based on 353 age-associated CpG sites and facilitates good precision of age-estimation in various tissue types. The relatively high precisions of age-predictions hold high hopes in forensics to estimate the donor age of blood traces or of people with allegedly unknown age. Furthermore, epigenetic age-predictions do not only correlate with chronological age, they are also indicative for life expectancy (Marioni et al., 2015, 2018; Field et al., 2018). Particularly specific hypomethylated CpGs seem to correlate with all-cause mortality (Lin et al., 2016). This indicates that epigenetic age-predictors may be trained to rather correlate with biological age (Levine et al., 2018).

Epigenetic age-predictions are less precise for cells in culture, due to the above mentioned impact of *in vitro* expansion on the epigenetic makeup (Horvath, 2013; Frobel et al., 2014; Sheng et al., 2018). There is a moderate correlation between age-associated and senescence-associated DNAm changes (Bork et al., 2010; Pasumarthy et al., 2017) and hence it is not surprising that several epigenetic clocks for aging are also increasing with higher passage numbers (Horvath et al., 2018). However, as mentioned above, replicative senescence and aging are overall rather reflected by independent CpGs, indicating that the different types of epigenetic clocks might be modulated independently.

## Resetting of Epigenetic Clocks by Reprogramming Into iPSCs

It is possible to rejuvenate cells by reprogramming into induced pluripotent stem cells (iPSCs). While in pluripotent state, iPSCs can be passaged virtually infinitively without any signs of cellular aging. Furthermore, iPSCs derived from elderly organisms can give rise to young organisms that pursue normal aging, as for example demonstrated in mice (Boland et al., 2009). Also on molecular level iPSCs seem to be fully rejuvenated: their telomeres become elongated and other age-related molecular parameters are reset upon reprogramming (Marion et al., 2009). Notably, age-associated as well as senescence-associated DNAm patterns are completely reversed in iPSCs (Horvath, 2013; Koch et al., 2013; Weidner et al., 2014). This epigenetic switch apparently occurs simultaneous with DNAm changes in pluripotency associated CpGs, indicating that the epigenetic rejuvenation belongs to the fundamental changes in reprogramming (Franzen et al., 2018). However, it has also been demonstrated that the loss of cell-type specific gene expression, e.g., for fibroblast lineage, follows different kinetics, which may suggest that there might be a safe time window for rejuvenation without complete erasure of somatic identity (Olova et al., 2018).

Upon re-differentiation of iPSCs toward other cell types the senescence-associated DNAm patterns are continuously reacquired as observed during culture expansion of primary cells (Frobel et al., 2014). In contrast, the estimates for epigenetic age remain overall rejuvenated in the iPSC-derived cells and are only very slowly accelerated upon differentiation (Frobel et al., 2014). Direct conversion of cells, e.g., into induced neurons (iNs), at least initially retains age-associated transcriptomic and epigenetic signatures (Mertens et al., 2015; Huh et al., 2016). Furthermore, direct conversion into induced neuronal stem cells (iNSCs) maintained some of the age-related DNAm patterns, which further erode upon culture expansion (Sheng et al., 2018). This epigenetic rejuvenation of iPSC and iPSC-derived cells needs to be taken into account when studying age-related diseases – to better address such research questions there is a need to identify ways to artificially accelerate epigenetic aging clocks.

To investigate if overexpression of the catalytic subunit of the human telomerase (TERT) would be enough to stop aging, we have immortalized fibroblasts and MSCs with TERT. While this facilitated elongation of telomeres and long-term expansion without any signs of replicative senescence, it did not reset senescence-associated DNAm changes – in fact, these epigenetic modifications were even further acquired during culture expansion of immortalized lines (Koch et al., 2013). This was more recently validated also for age-associated DNAm changes (Kabacik et al., 2018; Lu et al., 2018). Furthermore, age-associated DNAm changes were not generally accelerated in telomeropathies, such as dyskeratosis congenita (Weidner et al., 2016). Thus, TERT and telomere length have apparently no immediate impact on the epigenetic clock, but this may be further analyzed in the future.

## How are Epigenetic Clocks Regulated?

So far, it is largely unclear how the very complex DNAm patterns can be modulated during development by predominantly two *de novo* DNA methyltransferases: DNMT3A and DNMT3B. We have recently demonstrated that different splice variants of *DNMT3A* have transcript specific effects on the DNAm pattern (Bozic et al., 2018), but it remains unclear how these enzymes are guided to specific sites in the genome. It is conceivable that this process is mediated by other DNA-binding proteins or long non-coding RNAs. Various transcription factor motifs have been associated with senescence-associated DNAm changes, including binding sites for early growth response protein 1 (EGR1), activating enhancer-binding protein 2 (TFAP2A), protein C-ets-1 (ETS1), neuroblastoma MYC oncogene (MYCN), and aryl hydrocarbon receptor nuclear translocator (ARNT) (Hänzelmann et al., 2015; Pasumarthy et al., 2017). Furthermore, the long non-coding RNA *HOTAIR* was suggested to target such differentially methylated regions, potentially by triple helix formation (Kalwa et al., 2016).

On the other hand, there is also evidence that the differentially methylated regions of epigenetic clocks are not directly mediated. We have used bisulfite barcoded amplicon sequencing (BBA-seq) to compare senescence-associated DNAm in different subpopulations of MSCs. Notably, in clonally derived subpopulations, the DNAm levels of neighboring CpGs differed extensively, indicating that these genomic regions are not synchronously modified during senescence (Franzen et al., 2017). In a more recent study, we have analyzed if senescence-associated DNAm changes are strand-specific by BBA-seq of hairpin-linked DNA molecules. In fact, many CpG dyads at these sites became only methylated on either the forward or the reverse strand. This hemimethylation was conserved over many passages, indicating that it was not due to insufficient maintenance of DNAm (Franzen et al., 2018). Circular chromatin conformation capture (4C) of senescence-associated CpGs indicated that there is no specific interaction of these genomic regions with other regions that undergo senescence-associated DNAm changes (Franzen et al., 2018). Furthermore, functional annotation of age-associated CpGs showed enrichment in CCCTC-binding factor (CTCF), which is relevant for the 3D organization of the genome (Day et al., 2013; Wang et al., 2018). It appears that the changes in DNAm partly overlap with changing histone modifications upon aging (Johansson et al., 2013). These findings suggest that senescence-associated and age-associated DNAm are not regulated in a targeted manner but rather evoked by other chromatin modifications.

## What is the Reason for Epigenetic Aging?

It is still controversially discussed if aging is governed by a purposeful program or if it rather resembles an accumulation of stochastic, accidental events (Hayflick, 2007). While several authors argued that aging cannot be programmed (Blagosklonny, 2013), there is also evidence that it may be evolutionary purposeful for the species (Longo et al., 2005; Mitteldorf, 2016; Horvath and Raj, 2018). Aging necessitates a regular generation cycle, which supports better adaptation to environmental changes. In analogy, replicative senescence has been suggested to simply resemble an artificial process caused by the cell culture conditions, or to reflect a beneficial organized process that is somewhat related to aging. It has also been postulated that replicative senescence acts as a safeguard for malignant transformation (Campisi, 2000). On the other hand, there seems to be a significant overlap of DNAm changes in senescence and cancer (Cruickshanks et al., 2013), indicating that senescence might even promote malignant transformation. Furthermore, fibroblasts surrounding the tumor are known drivers of tumor growth by providing a permissive environment (Campisi, 2005) and hence senescence of neighboring cells might also be relevant. However, despite global similarities there are locally distinct DNAm changes in senescence versus malignant transformation (Xie et al., 2018). Overall, the precision of epigenetic aging clocks is very low in cancer, which might be due to the fact that cancer cells capture only the specific epigenetic state of the tumor-initiating cell (Lin and Wagner, 2015; Eipel et al., 2019). Most types of malignancies occur in the elderly and it may hence be speculated, that the chromatin reorganization upon aging supports specific mutations – age-associated DNAm changes would then rather resemble a trigger than a safeguard for malignant transformation (Wagner et al., 2015).

## Conclusion

Epigenetic clocks for aging and senescence are certainly ticking independently, but the striking similarities indicate that the processes are regulated by similar means. There is evidence that the epigenetic modifications are not governed by targeting of regulatory complexes but that the DNAm changes may rather be orchestrated indirectly by other types of chromatin organization. Studying of senescence-associated changes *in vitro* has several strategic advantages for aging research: Effects of relevant genes or drugs can be systematically screened in a high throughput manner and with large compound libraries. It appears to be plausible that such findings can then be extrapolated to understand how to modulate age-associated epigenetic modifications – and possibly the process of aging.

## Author Contributions

The author confirms being the sole contributor of this work and has approved it for publication.

## Conflict of Interest Statement

WW is cofounder of Cygenia GmbH (www.cygenia.com), which can provide service for Epigenetic Senescence Signatures and Epigenetic Aging Signatures to other scientists.
